# Whole genome and phylogenetic analysis of two SARS-CoV-2 strains isolated in Italy in January and February 2020: additional clues on multiple introductions and further circulation in Europe

**DOI:** 10.2807/1560-7917.ES.2020.25.13.2000305

**Published:** 2020-04-02

**Authors:** Paola Stefanelli, Giovanni Faggioni, Alessandra Lo Presti, Stefano Fiore, Antonella Marchi, Eleonora Benedetti, Concetta Fabiani, Anna Anselmo, Andrea Ciammaruconi, Antonella Fortunato, Riccardo De Santis, Silvia Fillo, Maria Rosaria Capobianchi, Maria Rita Gismondo, Alessandra Ciervo, Giovanni Rezza, Maria Rita Castrucci, Florigio Lista, on behalf of ISS COVID-19 study group

**Affiliations:** 1 Department of Infectious Diseases, Istituto Superiore di Sanità, Rome, Italy; 2 Scientific Department Army Medical Center, Rome, Italy; 3 These authors contributed equally to the work; 4 Laboratory of Virology, National Institute for Infectious Diseases “Lazzaro Spallanzani” IRCCS, Rome, Italy; 5 Clinical Microbiology, Virology and Bioemergency, L. Sacco University Hospital, Milan, Italy; 6 The members of the ISS COVID-19 study group are acknowledged at the end of the article

**Keywords:** SARS-CoV-2, Whole genome sequence, phylogenetic analysis, Europe

## Abstract

Whole genome sequences of SARS-CoV-2 obtained from two patients, a Chinese tourist visiting Rome and an Italian, were compared with sequences from Europe and elsewhere. In a phylogenetic tree, the Italian patient’s sequence clustered with sequences from Germany while the tourist’s sequence clustered with other European sequences. Some additional European sequences in the tree segregated outside the two clusters containing the patients’ sequences. This suggests multiple SARS-CoV-2 introductions in Europe or virus evolution during circulation.

An outbreak of a viral respiratory illness (officially named by the World Health Organization coronavirus disease, COVID-19) caused by the newly discovered severe acute respiratory syndrome coronavirus (SARS-CoV-2), started around mid-December 2019, in the city of Wuhan, Hubei province, China [1]. The outbreak subsequently spread further and as at 31 March 2020, 750,890 cases of COVID-19 have been confirmed worldwide including 668,345 outside China [2]. Since 20 February 2020, sustained local transmission has been documented in Italy [3], where to date, 98,716 COVID-19 cases testing positive for SARS-CoV-2 have been diagnosed, with 10,943 deaths [4].

To gain further understanding on the molecular epidemiology of the outbreak in Italy, we characterised the full-genome sequence of two SARS-CoV-2 strains respectively isolated from two patients diagnosed in the country. The first patient was a Chinese tourist from Wuhan diagnosed at the end of January, who had visited Rome and not been in areas of Italy later found to be the initially affected areas of the epidemic in Lombardy. The second patient was an Italian person, with no apparent direct epidemiological link with China and who was diagnosed in the second half of February in Lombardy. The sequences presented are analysed in the context of other available genome sequences from Europe and elsewhere.

## Patients, virus cultivation and whole genome sequencing

The two patients in this study had both been hospitalised with an acute respiratory illness (pneumonia), showing a bilateral lung involvement with ground-glass opacity, requiring intensive care. The Chinese patient had had onset of symptoms on 29 January 2020 and had been diagnosed in a hospital in Rome. The Italian patient whose onset of symptoms had occurred on 10 February 2020 had been diagnosed in a hospital in Milan. Biological samples from both patients had been confirmed as being SARS-CoV-2 positive by the National Reference Laboratory (NRL) of the Istituto Superiore di Sanità (ISS) in Rome.

The samples used for this study were nasopharyngeal swabs. These had been respectively sampled on the same day of hospitalisation, when symptoms occurred, for the Chinese tourist and 10 days after symptom onset for the Italian patient. An aliquot of each patient’s nasopharyngeal sample was used to generate in vitro cultures in Vero cells grown in modified Eagle’s medium (MEM; Gibco, Thermofisher, United Kingdom) supplemented with GlutaMAX. A total of 140 µL of each culture’s supernatant was used for viral RNA extraction using the QIAMP VIRAL RNA mini kit (Qiagen, Hilden, Germany). The obtained genomic RNAs were retro-transcribed using the SuperScript III Reverse Transcriptase kit (Invitrogen, Carisbad, United States (US)) and double-stranded DNAs were subsequently obtained by Klenow enzyme (Roche, Basel, Switzerland) according to the manufacturer’s instructions. The Nextera XT kit was used for library preparations and whole genome sequencing was performed using the Illumina Miseq Reagent Nano Kit, V2 (2 x 150 cycles) on the Illumina MiSeq instrument (Illumina, San Diego, US). The reads were trimmed for quality and length and assembled by mapping to the reference genome from Wuhan, China (GenBank accession number: NC_045512.2) using Geneious Prime (www.geneious.com) [5]. Viral sequences from the two patients were deposited in the Global Initiative on Sharing All Influenza Data (GISAID; https://www.gisaid.org/epiflu-applications/next-hcov-19-app/ ).

## Phylogenetic analysis

To analyse the obtained SARS-CoV-2 genomes respectively derived from the infected Chinese tourist (GISAID accession ID: EPI_ISL_412974) and the Italian patient (GISAID accession ID: EPI_ISL_412973) in a phylogenetic context, a dataset of 40 available SARS-Cov-2 complete genomes from different countries was retrieved from GISAID (https://www.gisaid.org/, last access 2 March 2020; Supplementary material). Sequence alignment was performed using MUltiple Sequence Comparison by Log- Expectation (MUSCLE) software (http://www.clustal.org) [6]. Estimation of the best fitting substitution model (Hasegawa, Kishino, and Yano, HKY model) and inference of the phylogenetic tree were conducted by a maximum likelihood approach using Molecular Evolutionary Genetics Analysis across Computing Platforms (MEGA X; https://www.megasoftware.net/) [7]. Support for the tree topology was estimated with 1,000 bootstrap replicates.

The maximum likelihood phylogenetic tree in the Figure shows a main clade containing several clusters. The viral genome sequence of the Chinese tourist (GISAID accession ID: EPI_ISL_412974) was identical to that retrieved from one sample of another Chinese tourist, hospitalised at the same hospital in Rome (GISAID accession ID: EPI_ISL_410546). The latter was closely related to that of another sample taken from the same patient (GISAID accession ID: EPI_ISL_410545). These three genome sequences were located in a cluster with genomes mainly from Europe (England, France, Italy, Sweden), but also one from Australia (Figure, highlighted in dark red).

**Figure:**
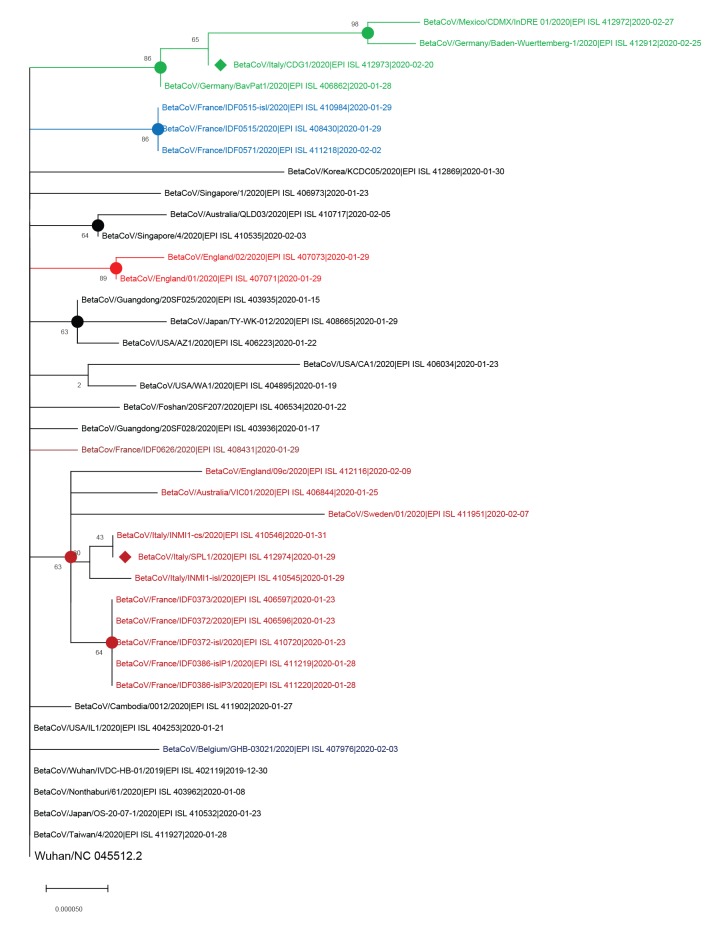
Phylogenetic analysis of two SARS-CoV-2 complete genome sequences retrieved in this study, with available complete sequences from different countries^a^ (n = 40 genome sequences)

GISAID: Global Initiative on Sharing All Influenza Data; HKY: Hasegawa, Kishino, and Yano; MEGA X: Molecular Evolutionary Genetics Analysis across Computing Platforms; SARS-CoV-2: severe acute respiratory syndrome coronavirus.

Main clusters are highlighted in different colours. The Wuhan reference genome is in larger font (GenBank accession number: NC_045512.2). The filled circles represent the main supported clusters (bootstrap support values are indicated at the level of the nodes). The scale bar at the bottom of the tree represents 0.000050 nt substitutions per site. The cluster containing the viral sequence of the Chinese tourist who had visited Rome, Italy (GISAID accession ID: EPI_ISL_412974) is in dark red. This cluster includes viral sequences derived from two samples (sputum and nasopharyngeal swabs) of another Chinese tourist visiting Rome (GISAID accession IDs: EPI_ISL_410545 and EPI_ISL_410546). The viral genome sequence (GISAID accession ID: EPI_ISL_412973) derived from a patient from Lombardy, Italy, is in a cluster highlighted in green, which is different from that containing the Chinese tourist’s sequence.

^a ^The tree wasbuilt by using the best fitting substitution model (HKY) through MEGA X software.

The genome sequence from the Italian patient in Lombardy (EPI_ISL_412973) appeared in contrast to be located in a different cluster including two genome sequences from Germany (EPI_ISL_406862 Bavaria/Munich and EPI_ISL_412912 Baden-Wuerttemberg-1) and one genome sequence from Mexico (EPI_ISL_ 412972), (Figure, highlighted in green).

In the tree, some sequences from other SARS-CoV-2 collected in Europe segregated in separate clusters from the two clusters containing the respective patient sequences characterised in this study. There was for example a cluster formed by two sequences from England and a cluster formed by three sequences from France.

Using an alignment, the single nt polymorphisms (SNPs) composition and the potentially resulting variable amino-acids in derived protein sequences compared with the Wuhan reference sequences (MN908947 and NC_045512), were investigated for the genome sequences retrieved in this study, as well as three other genome sequences (EPI_ISL_412972, EPI_ISL_ 412912, EPI_ISL_406862) that clustered with the sequence of the patient in Lombardy.

The genome-wide SNPs are reported in Table 1 (positions referred respect to the reference sequence; GenBank accession number: NC_045512). The corresponding amino-acid positions and variations inside the proteins are shown in Table 2.

**Table 1:** Single nt polymorphisms (SNPs)^a^ deduced by comparison of two whole genome sequences of SARS-CoV-2 characterised in this study^b^ with selected SARS-CoV-2 sequences (n = 7 compared sequences)

SARS-CoV-2 sequence ID (country from which the sequence originated)	241	3037	10265	11083	13206	14408	15806	23403	26144	28881	28882	28883
	5' UTR	ORF1ab gene	ORF1ab gene	ORF 1ab gene	ORF1ab gene	ORF1ab gene	ORF1ab gene	Gene S	ORF3a gene	Gene N	Gene N	Gene N
**NC_045512 (China)**	C	C	G	G	C	C	A	A	G	G	G	G
**MN908947 (China)**	C	C	G	G	C	C	A	A	G	G	G	G
**EPI_ISL:412972 (Mexico)**	T	T	G	G	G	T	-	G	G	A	A	C
**EPI_ISL: 412912 (Germany)**	T	T	A	G	C	T	A	G	G	A	A	C
**EPI_ISL: 406862 (Germany)**	T	T	G	G	C	C	A	G	G	G	G	G
**EPI_ISL_412973 (Italy)**	T	T	G	G	C	T	A	G	G	G	G	G
**EPI_ISL_412974 (Italy)**	C	C	G	T	C	C	A	A	T	G	G	G

N: nucleocapsid protein; ORF: open reading frame; ORF1ab: ORF encoding polyprotein; S: surface glycoprotein; SARS-CoV-2: severe acute respiratory syndrome coronavirus; SNP: single nt polymorphism; UTR: untranslated region.

^a ^SNPs are shown according to nt positions in the genome sequence and gene location.

^b ^The two sequences characterised in this study are the ones from Italy (EPI_ISL_412973 and EPI_ISL_412974).

**Table 2:** Amino acid variations^a^ deduced by comparing translations of two whole genome sequences of SARS-CoV-2 characterised in this study^b^ with those of selected SARS-CoV-2 sequences (n = 7 compared sequences)

SARS-CoV-2 strains	924	3334	3606	4314	4704	5170	614	251	203	204
	ORF1ab	ORF1ab	ORF1ab	ORF1ab	ORF1ab	ORF1ab	Surface glycoprotein	ORF3a	Nucleocapsid phosphoprotein	Nucleocapsid phosphoprotein
**NC_045512 (China)**	F	G	L	A	P	Q	D	G	R	G
**MN908947 (China)**	F	G	L	A	P	Q	D	G	R	G
**EPI_ISL:412972 (Mexico)**	F	G	L	G	L	-^c^	G	G	K	R
**EPI_ISL: 412912 (Germany)**	F	S	L	A	L	Q	G	G	K	R
**EPI_ISL: 406862 (Germany)**	F	G	L	A	P	Q	G	G	R	G
**EPI_ISL_412973 (Italy)**	F	G	L	A	L	Q	G	G	R	G
**EPI_ISL_412974 (Italy)**	F	G	F	A	P	Q	D	V	R	G

ORF: open reading frame; ORF1ab: ORF encoding polyprotein; SARS-CoV-2: severe acute respiratory syndrome coronavirus.

^a^ The amino acid positions refer to those in each respective protein sequence of the Wuhan reference (GenBank accession number: MN908947), starting from the first methionine.

^b ^The two sequences characterised in this study are the ones from Italy (EPI_ISL_412973 and EPI_ISL_412974).

^c^ -: possible sequencing error.

The genome sequence from the Chinese tourist hospitalised in Rome differed in two nt positions from that of the COVID-19 patient in Wuhan (NC_045512), while the genome sequence isolated from the Italian patient showed four nt variations (Table 1). 

For the sequence of the Chinese tourist, the first SNP inside ORF1ab (bps 3037, AA 924) did not result in an amino acid change.

In the Table 2 that depicts five sequences characterised outside of China, overall eight missense mutations can be observed compared to the two reference Wuhan sequences: four locate to the ORF1ab polyprotein, whereby only the mutation L3606F has previously been reported by Phan, 2020 [8]; one, D614G, locates to the surface glycoprotein and has been prior observed [8], but is not in the receptor binding domain (RDB), responsible for virus entry into host cell; one is in the ORF3a protein and two are in the nucleocapsid protein.

The sequence of the Chinese tourist hospitalised in Rome on 29 January (EPI_ISL_412974) presented a mutation 3606F in ORF1ab with respect to the reference Wuhan genome (L). In ORF3a, this sequence had a V at amino acid position 251, as opposed to a G in the references from Wuhan.

Meanwhile, the sequence of the Italian patient from Lombardy (EPI_ISL 412973) presented an L at amino acidic position 4704 with respect to the reference Wuhan genome (P). It also had a mutation in the surface glycoprotein, at amino acidic position 614, where it showed a G compared to the reference sequences from Wuhan that presented a D at that position.

With regard to the nucleocapsid protein, both of the sequences from the Italian patient and Chinese tourist presented the same amino acids of the references Wuhan genomes.

## Discussion

In this study, the full length genomes of two SARS-CoV-2 strains (EPI_ISL_412973 and EPI_ISL_412974) isolated in Italy, one from an Italian patient, the other from a Chinese tourist visiting Rome, are completely sequenced and analysed, after virus cultivation. Compared to the viral genome sequence of the COVID-19 patient in Wuhan, the sequence from the Chinese tourist had two nt differences, while that of the Italian patient had four. Phylogenetic analysis consistently placed the Italian patient’s strain in a distinct cluster from the tourist’s strain. The strain of the Italian patient grouped with other viral strains identified in Germany and Mexico, while the strain from the Chinese tourist, related with the Wuhan virus strain, clustered with different European strains and a strain from Australia. Other sequences from strains collected in Europe, which were included in the phylogenetic analysis, ended up in separate clusters from the ones respectively containing the sequences of the two patients reported here. The results are consistent with several introductions of SARS-CoV-2 in Europe and/or further circulation of the single strain originating in Wuhan with concurrent evolution and accumulation of mutations. The mutations found in the virus identified in Lombardy, compared with the reference Wuhan strain, and the identification of amino acids changes, should be further investigated to understand whether they may affect virus characteristics. Some limitations need be mentioned: first, the lack of epidemiological information available with most sequences deposited in the database; second, the number of genomes available at the time of the analysis and consequently their selection. Nevertheless, these data may be useful to understand the dynamics of the local transmission of SARS-CoV-2 in Europe.

## Supplementary Data

204000 Supplementary Material

## References

[1] Wu P Hao X Lau EHY Wong JY Leung KSM Wu JT Real-time tentative assessment of the epidemiological characteristics of novel coronavirus infections in Wuhan, China, as at 22 January 2020. Euro Surveill. 2020;25(3):25. 10.2807/1560-7917.ES.2020.25.3.2000044 31992388 PMC6988272

[2] World Health Organization (WHO). Coronavirus disease 2019 (COVID-19) Situation Report – 71. Geneva: WHO; 31 Mar 2020. Available from: https://www.who.int/docs/default-source/coronaviruse/situation-reports/20200331-sitrep-71-covid-19.pdf?sfvrsn=4360e92b_6

[3] European Centre for Disease Control and Prevention (ECDC). Coronavirus disease 2019 (COVID-19) pandemic: increased transmission in the EU/EEA and the UK – seventh update, 25 March 2020. Stockholm: ECDC; 2020. Available from: https://www.ecdc.europa.eu/sites/default/files/documents/RRA-seventh-update-Outbreak-of-coronavirus-disease-COVID-19.pdf

[4] Epicentro, Epidemiology for public health, Istituto Superiore di Sanità (ISS). Sorveglianza Integrata COVID-19 in Italia. Aggiornamento 31 marzo 2020. [COVID-19 Integrated Surveillance in Italy. Update 31 March 2020]. Rome: ISS; 31 Mar 2020. Italian. Available from: https://www.epicentro.iss.it/coronavirus/bollettino/Infografica_31marzo%20ITA.pdf

[5] Kearse M Moir R Wilson A Stones-Havas S Cheung M Sturrock S Geneious Basic: an integrated and extendable desktop software platform for the organization and analysis of sequence data. Bioinformatics. 2012;28(12):1647-9. 10.1093/bioinformatics/bts199 22543367 PMC3371832

[6] Edgar RC MUSCLE: a multiple sequence alignment method with reduced time and space complexity. BMC Bioinformatics. 2004;5(1):113. 10.1186/1471-2105-5-113 15318951 PMC517706

[7] Kumar S Stecher G Li M Knyaz C Tamura K MEGA X: Molecular Evolutionary Genetics Analysis across computing platforms. Mol Biol Evol. 2018;35(6):1547-9. 10.1093/molbev/msy096 29722887 PMC5967553

[8] Phan T Genetic diversity and evolution of SARS-CoV-2. Infect Genet Evol. 2020;81:104260. 10.1016/j.meegid.2020.104260 32092483 PMC7106203

