# Supplementary material for: Whole genome and phylogenetic analysis of two SARS-CoV-2 strains isolated in Italy in January and February 2020: additional clues on multiple introductions and further circulation in Europe
Source: Euro Surveill. 2020 Apr 2;25(13):2000305. doi: 10.2807/1560-7917.ES.2020.25.13.2000305 (PMC7140597; doi:10.2807/1560-7917.ES.2020.25.13.2000305)
Supplement: Supplementary Material [file 2000305_STEFANELLI_Supplementary_Material.pdf]

Supplementary Material

This supplementary material is hosted by *Eurosurveillance* as supporting information alongside the article [Whole genome and phylogenetic analysis of two SARS-CoV-2 strains isolated in Italy in January and February 2020: additional clues on multiple introductions and further circulation in Europe], on behalf of the authors, who remain responsible for the accuracy and appropriateness of the content. The same standards for ethics, copyright, attributions and permissions as for the article apply. Supplements are not edited by *Eurosurveillance* and the journal is not responsible for the maintenance of any links or email addresses provided therein.

*We gratefully acknowledge the Authors, the Originating and Submitting Laboratories for their sequence and metadata shared through GISAID, on which this research is based. All submitters of data may be contacted directly via **www.gisaid.org.***

- EPI_ISL_402119 BetaCoV/Wuhan/IVDC-HB-01/2019 Asia / China / Hubei / Wuhan 2019-12-30 National Institute for Viral Disease Control and Prevention, China CDC National Institute for Viral Disease Control and Prevention, China CDC Wenjie Tan, Xiang Zhao, Wenling Wang, Xuejun Ma, Yongzhong Jiang, Roujian Lu, Ji Wang, Weimin Zhou, Peihua Niu, Peipei Liu, Faxian Zhan, Weifeng Shi, Baoying Huang, Jun Liu, Li Zhao, Yao Meng, Xiaozhou He, Fei Ye, Na Zhu, Yang Li, Jing Chen, Wenbo Xu, George F. Gao, Guizhen Wu

- EPI_ISL_403935 BetaCoV/Guangdong/20SF025/2020 Asia / China / Guangdong / Shenzhen 2020-01-15 Guangdong Provincial Center for Diseases Control and Prevention; Guangdong Provincial Public Health Department of Microbiology, Guangdong Provincial Center for Diseases Control and Prevention Min Kang, Jie Wu, Jing Lu, Tao Liu, Baisheng Li, Shujiang Mei, Feng Ruan, Lifeng Lin, Changwen Ke, Haojie Zhong, Yingtao Zhang, Lirong Zou, Xuguang Chen, Qi Zhu, Jianpeng Xiao, Jianxiang Geng, Zhe Liu, Jianxiong Hu, Weilin Zeng, Xing Li, Yuhuang Liao, Xiujuan Tang, Songjian Xiao, Ying Wang, Yingchao Song, Xue Zhuang, Lijun Liang, Guanhao He, Huihong Deng, Tie Song, Jianfeng He, Wenjun Ma

- EPI_ISL_403962 BetaCoV/Nonthaburi/61/2020 Asia / Thailand / Nonthaburi 2020-01-08 Bamrasnaradura Hospital 1. Department of Medical Sciences, Ministry of Public Health, Thailand 2. Thai Red Cross Emerging Infectious Diseases - Health Science Centre 3. Department of Disease Control, Ministry of Public Health, Thailand Pilailuk,Okada; Siripaporn,Phuygun; Thanutsapa,Thanadachakul; Supaporn,Wacharapluesadee; Sittiporn,Parnmen; Warawan,Wongboot; Sunthareeya,Waicharoen; Rome,Buathong; Malinee,Chittaganpitch; Nanthawan,Mekha

- EPI_ISL_403936 BetaCoV/Guangdong/20SF028/2020 Asia / China / Guangdong / Zhuhai 2020-01-17 Guangdong Provincial Center for Diseases Control and Prevention; Guangdong Provincial Public Health Department of Microbiology, Guangdong Provincial Center for Diseases Control and Prevention Min Kang, Jie Wu, Jing Lu, Tao Liu, Baisheng Li, Shujiang Mei, Feng Ruan, Lifeng Lin, Changwen Ke, Haojie Zhong, Yingtao Zhang, Lirong Zou, Xuguang Chen, Qi Zhu, Jianpeng Xiao, Jianxiang Geng, Zhe Liu, Jianxiong Hu, Weilin Zeng, Xing Li, Yuhuang Liao, Xiujuan Tang, Songjian Xiao, Ying Wang, Yingchao Song, Xue Zhuang, Lijun Liang, Guanhao He, Huihong Deng, Tie Song, Jianfeng He, Wenjun Ma

- EPI_ISL_404895 BetaCoV/USA/WA1/2020 North America / USA / Washington / Snohomish County 2020-01-19 Providence Regional Medical Center Division of Viral Diseases, Centers for Disease Control and Prevention Queen,K., Tao,Y., Li,Y., Paden,C.R., Lu,X., Zhang,J., Gerber,S.I., Lindstrom,S., Tong,S.

- EPI_ISL_404253 BetaCoV/USA/IL1/2020 North America / USA / Illinois / Chicago 2020-01-21 IL Department of Public Health Chicago Laboratory Pathogen Discovery, Respiratory Viruses Branch, Division of Viral Diseases, Centers for Dieases Control and Prevention Ying Tao, Krista Queen, Clinton R. Paden, Jing Zhang, Yan Li, Anna Uehara, Xiaoyan Lu, Brian Lynch, Senthil Kumar K. Sakthivel, Brett L. Whitaker, Shifaq Kamili, Lijuan Wang, Janna' R. Murray, Susan I. Gerber, Stephen Lindstrom, Suxiang Tong

- EPI_ISL_406034 BetaCoV/USA/CA1/2020 North America / USA / California / Los Angeles 2020-01-23 California Department of Public Health Pathogen Discovery, Respiratory Viruses Branch, Division of

Viral Diseases, Centers for Dieases Control and Prevention Anna Uehara, Krista Queen, Ying Tao, Yan Li, Clinton R. Paden, Jing Zhang, Xiaoyan Lu, Brian Lynch, Senthil Kumar K. Sakthivel, Brett L. Whitaker, Shifaq Kamili, Lijuan Wang, Janna' R. Murray, Susan I. Gerber, Stephen Lindstrom, Suxiang Tong

- EPI_ISL_406223 BetaCoV/USA/AZ1/2020 North America / USA / Arizona / Phoenix 2020-01-22 Arizona Department of Health Services Pathogen Discovery, Respiratory Viruses Branch, Division of Viral Diseases, Centers for Disease Control and Prevention Ying Tao, Clinton R. Paden, Krista Queen, Anna Uehara, Yan Li, Jing Zhang, Xiaoyan Lu, Brian Lynch, Senthil Kumar K. Sakthivel, Brett L. Whitaker, Shifaq Kamili, Lijuan Wang, Janna' R. Murray, Susan I. Gerber, Stephen Lindstrom, Suxiang Tong

- EPI_ISL_406534 BetaCoV/Foshan/20SF207/2020 Asia / China / Guangdong / Foshan 2020-01-22 Guangdong Provincial Center for Diseases Control and Prevention; Guangdong Provincial Public Health Guangdong Provincial Center for Diseases Control and Prevention Min Kang, Jie Wu, Jing Lu, Tao Liu, Baisheng Li, Shujiang Mei, Feng Ruan, Lifeng Lin, Changwen Ke, Haojie Zhong, Yingtao Zhang, Lirong Zou, Xuguang Chen, Qi Zhu, Jianpeng Xiao, Jianxiang Geng, Zhe Liu, Jianxiong Hu, Weilin Zeng, Xing Li, Yuhuang Liao, Xiujuan Tang, Songjian Xiao, Ying Wang, Yingchao Song, Xue Zhuang, Lijun Liang, Guanhao He, Huihong Deng, Tie Song, Jianfeng He, Wenjun Ma

- EPI_ISL_406597 BetaCoV/France/IDF0373/2020 Europe / France / Ile-de-France / Paris 2020-01-23 Department of Infectious and Tropical Diseases, Bichat Claude Bernard Hospital, Paris National Reference Center for Viruses of Respiratory Infections, Institut Pasteur, Paris Mélanie Albert, Marion Barbet, Sylvie Behillil, Méline Bizard, Angela Brisebarre, Flora Donati, Vincent Enouf, Maud Vanpeene, Sylvie van der Werf, Yazdan Yazdanpanah, Xavier Lescure.

- EPI_ISL_406596 BetaCoV/France/IDF0372/2020 Europe / France / Ile-de-France / Paris 2020-01-23 Department of Infectious and Tropical Diseases, Bichat Claude Bernard Hospital, Paris National Reference Center for Viruses of Respiratory Infections, Institut Pasteur, Paris Mélanie Albert, Marion Barbet, Sylvie Behillil, Méline Bizard, Angela Brisebarre, Flora Donati, Vincent Enouf, Maud Vanpeene, Sylvie van der Werf, Yazdan Yazdanpanah, Xavier Lescure.

- EPI_ISL_406844 BetaCoV/Australia/VIC01/2020 Oceania / Australia / Victoria / Clayton 2020-01-25 Monash Medical Centre Collaboration between the University of Melbourne at The Peter Doherty Institute for Infection and Immunity, and the Victorian Infectious Disease Reference Laboratory Caly,L., Seemann,T., Schultz,M., Druce,J. and Taiaroa,G

- EPI_ISL_406862 BetaCoV/Germany/BavPat1/2020 Europe / Germany / Bavaria / Munich 2020-01-28 Charité Universitätsmedizin Berlin, Institute of Virology; Institut für Mikrobiologie der Bundeswehr, Munich Charité Universitätsmedizin Berlin, Institute of Virology Victor M Corman, Julia Schneider, Talitha Veith, Barbara Mühlemann, Markus Antwerpen, Christian Drosten, Roman Wölfel

- EPI_ISL_406973 BetaCoV/Singapore/1/2020 Asia / Singapore 2020-01-23 Singapore General Hospital National Public Health Laboratory Mak, TM; Octavia S; Chavatte JM; Zhou, ZY; Cui, L; Lin, RTP

- EPI_ISL_407071 BetaCoV/England/01/2020 Europe / England 2020-01-29 Respiratory Virus Unit, Microbiology Services Colindale, Public Health England Respiratory Virus Unit, Microbiology Services Colindale, Public Health England Monica Galiano, Shahjahan Miah, Richard Myers, Angie Lackenby, Omolola Akinbami, Tiina Talts, Leena Bhaw, Kirstin Edwards, Jonathan Hubb, Joanna Ellis, Maria Zambon

- EPI_ISL_407073 BetaCoV/England/02/2020 Europe / England 2020-01-29 Respiratory Virus Unit, Microbiology Services Colindale, Public Health England Respiratory Virus Unit, Microbiology Services Colindale, Public Health England Monica Galiano, Shahjahan Miah, Richard Myers, Angie Lackenby, Omolola Akinbami, Tiina Talts, Leena Bhaw, Kirstin Edwards, Jonathan Hubb, Joanna Ellis, Maria Zambon.

- EPI_ISL_407976 BetaCoV/Belgium/GHB-03021/2020 Europe / Belgium / Leuven 2020-02-03 KU Leuven, Clinical and Epidemiological Virology KU Leuven, Clinical and Epidemiological Virology Bert Vanmechelen, Elke Wollants, Annabel Rector, Els Keyaerts, Lies Laenen, Marc Van Ranst, and Piet Maes

- EPI_ISL_408430 BetaCoV/France/IDF0515/2020 Europe / France / Ile-de-France / Paris 2020-01-29 Department of Infectious and Tropical Diseases, Bichat Claude Bernard Hospital, Paris National Reference Center for Viruses of Respiratory Infections, Institut Pasteur, Paris Mélanie Albert, Marion Barbet, Sylvie Behillil, Méline Bizard, Angela Brisebarre, Flora Donati, Vincent Enouf, Maud Vanpeene, Sylvie van der Werf, Yazdan Yazdanpanah, Xavier Lescure

- EPI_ISL_408431 BetaCov/France/IDF0626/2020 Europe / France / Ile-de-France / Paris 2020-01-29 Sorbonne Université, Inserm et Assistance Publique-Hôpitaux de Paris (Pitié Salpétrière) National Reference Center for Viruses of Respiratory Infections, Institut Pasteur, Paris Mélanie Albert, Marion Barbet, Sylvie Behillil, Méline Bizard, Angela Brisebarre, Flora Donati, Vincent Enouf, Maud Vanpeene, Sylvie van der Werf, Sonia Burrel, Anne-Geneviève Marcelin, Vincent Calvez, David Boutolleau, Elise Klément, Valérie Pourcher, Eric Caumes.

- EPI_ISL_408665 BetaCoV/Japan/TY-WK-012/2020 Asia / Japan / Tokyo 2020-01-29 Dept. of Virology III, National Institute of Infectious Diseases Pathogen Genomics Center, National Institute of Infectious Diseases Tsuyoshi Sekizuka, Shutoku Matsuyama, Naganori Nao, Kazuya Shirato, Makoto Takeda, Makoto Kuroda

- EPI_ISL_410532 BetaCoV/Japan/OS-20-07-1/2020 Asia / Japan / Osaka 2020-01-23 Dept. of Pathology, National Institute of Infectious Diseases Pathogen Genomics Center, National Institute of Infectious Diseases Tsuyoshi Sekizuka, Harutaka Katano, Shutoku Matsuyama, Naganori Nao, Kazuya Shirato, Motoi Suzuki, Hideki Hasegawa, Takaji Wakita, Makoto Takeda, Tadaki Suzuki, Makoto Kuroda

- EPI_ISL_410535 BetaCoV/Singapore/4/2020 Asia / Singapore 2020-02-03 National Centre for Infectious Diseases Programme in Emerging Infectious Diseases, Duke-NUS Medical School Danielle E Anderson, Martin Linster, Yan Zhuang, Jayanthi Jayakumar, David CB Lye, Yee Sin Leo, Barnaby E Young, Yvonne CF Su, Gavin JD Smith

- EPI_ISL_410545 BetaCoV/Italy/INMI1-isl/2020 Europe / Italy / Rome 2020-01-29 INMI Lazzaro Spallanzani IRCCS Laboratory of Virology, INMI Lazzaro Spallanzani IRCCS Maria R. Capobianchi, Cesare E. M. Gruber, Martina Rueca, Barbara Bartolini, Francesco Messina, Emanuela Giombini, Francesca Colavita, Concetta Castilletti, Eleonora Lalle, Fabrizio Carletti, Emanuele Nicastri, Giuseppe Ippolito.

- EPI_ISL_410546 BetaCoV/Italy/INMI1-cs/2020 Europe / Italy / Rome 2020-01-31 INMI Lazzaro Spallanzani IRCCS Laboratory of Virology, INMI Lazzaro Spallanzani IRCCS Maria R. Capobianchi, Cesare E. M. Gruber, Martina Rueca, Fabrizio Carletti, Barbara Bartolini, Francesco Messina, Emanuela Giombini, Francesca Colavita, Concetta Castilletti, Eleonora Lalle, Emanuele Nicastri, Giuseppe Ippolito.

- EPI_ISL_410717 BetaCoV/Australia/QLD03/2020 Oceania / Australia / Queensland / Gold Coast 2020-02-05 Pathology Queensland Public Health Virology Laboratory Ben Huang, Alyssa Pyke, Amanda De Jong, Andrew Van Den Hurk, Carmel Taylor, David Warrilow, Doris Genge, Elisabeth Gamez, Glen Hewitson, Ian Maxwell Mackay, Inga Sultana, Jamie McMahon, Jean Barcelon, Judy Northill, Mitchell Finger, Natalie Simpson, Neelima Nair, Peter Burtonclay, Peter Moore, Sarah Wheatley, Sean Moody, Sonja Hall-Mendelin, Timothy Gardam, and Frederick Moore.

- EPI_ISL_410720 BetaCoV/France/IDF0372-isl/2020 Europe / France / Ile-de-France / Paris 2020-01-23 Department of Infectious and Tropical Diseases, Bichat Claude Bernard Hospital, Paris National Reference Center for Viruses of Respiratory Infections, Institut Pasteur, Paris Mélanie Albert, Marion Barbet, Sylvie Behillil, Méline Bizard, Angela Brisebarre, Flora Donati, Vincent Enouf, Maud Vanpeene, Sylvie van der Werf, Yazdan Yazdanpanah, Xavier Lescure.

- EPI_ISL_410984 BetaCoV/France/IDF0515-isl/2020 Europe / France / Ile-de-France / Paris 2020-01-29 Department of Infectious and Tropical Diseases, Bichat Claude Bernard Hospital, Paris National Reference Center for Viruses of Respiratory Infections, Institut Pasteur, Paris Mélanie Albert, Marion Barbet, Sylvie Behillil, Méline Bizard, Angela Brisebarre, Flora Donati, Vincent Enouf, Maud Vanpeene, Sylvie van der Werf, Yazdan Yazdanpanah, Xavier Lescure

- EPI_ISL_411218 BetaCoV/France/IDF0571/2020 Europe / France / Ile-de-France / Paris 2020-02-02 Department of Infectious and Tropical Diseases, Bichat Claude Bernard Hospital, Paris Laboratoire Virpath, CIRI U111, UCBL1, INSERM, CNRS, ENS Lyon Olivier Terrier, Aurélien Traversier, Julien Fouret, Yazdan Yazdanpanah, Xavier Lescure, Catherine Legras-Lachuer, Alexandre Gaymard, Bruno Lina, Manuel Rosa-Calatrava

- EPI_ISL_411219 BetaCoV/France/IDF0386-islP1/2020 Europe / France / Ile-de-France / Paris 2020-01-28 Department of Infectious and Tropical Diseases, Bichat Claude Bernard Hospital, Paris Laboratoire Virpath, CIRI U111, UCBL1, INSERM, CNRS, ENS Lyon Olivier Terrier, Aurélien Traversier, Julien Fouret, Yazdan Yazdanpanah, Xavier Lescure, Alexandre Gaymard, Bruno Lina, Manuel Rosa-Calatrava

- EPI_ISL_411220 BetaCoV/France/IDF0386-islP3/2020 Europe / France / Ile-de-France / Paris 2020-01-28 Department of Infectious and Tropical Diseases, Bichat Claude Bernard Hospital, Paris Laboratoire Virpath, CIRI U111, UCBL1, INSERM, CNRS, ENS Lyon Olivier Terrier, Aurélien Traversier, Julien Fouret, Yazdan Yazdanpanah, Xavier Lescure, Alexandre Gaymard, Bruno Lina, Manuel Rosa-Calatrava

- EPI_ISL_411902 BetaCoV/Cambodia/0012/2020 Asia / Cambodia / Sihanoukville 2020-01-27 Virology Unit, Institut Pasteur du Cambodge. Virology Unit, Institut Pasteur du Cambodge (Sequencing done by: Jessica E Manning/Jennifer A Bohl at Malaria and Vector Research Research Laboratory, National Institute of Allergy and Infectious Diseases and Vida Ahyong from Chan-Zuckerberg Biohub) Erik A Karlsson, Jennifer A Bohl, Vida Ahyong, Veasna Duong, Philippe Dussart, Jessica E Manning.

- EPI_ISL_411927 BetaCoV/Taiwan/4/2020 Asia / Taiwan / Taipei 2020-01-28 Taiwan Centers for Disease Control Taiwan Centers for Disease Control Ji-Rong Yang, Yu-Chi-Lin, Jung-Jung Mu, Ming-Tsan-Liu

- EPI_ISL_411951 BetaCoV/Sweden/01/2020 Europe / Sweden 2020-02-07 unknown Unit for Laboratory Development and Technology Transfer, Public Health Agency of Sweden Bengner,M., Palmerus,M., Lindsjo,O., Lind Karlberg,M., Monteil,V., Appelberg,S., Brave,A., Muradrasoli,S. and Tegmark-Wisell,K.

- EPI_ISL_412116 BetaCoV/England/09c/2020 Europe / England 2020-02-09 Respiratory Virus Unit, Microbiology Services Colindale, Public Health England Respiratory Virus Unit, Microbiology Services Colindale, Public Health England Monica Galiano, Shahjahan Miah, Angie Lackenby, Omolola Akinbami, Tiina Talts, Leena Bhaw, Richard Myers, Steven Platt, Kirstin Edwards, Jonathan Hubb, Joanna Ellis, Maria Zambon

- EPI_ISL_412869 BetaCoV/Korea/KCDC05/2020 Asia / South Korea /Seoul 2020-01-30 Division of Viral Diseases, Center for Laboratory Control of Infectious Diseases, Korea Centers for Diseases Control and Prevention Division of Viral Diseases, Center for Laboratory Control of Infectious Diseases, Korea Centers for Diseases Control and Prevention Jeong-Min Kim, Yoon-Seok Chung, Namjoo Lee, Mi-Seon Kim, Sang Hee Woo, Hye-Jun Jo, Sehee Park, Heui Man Kim, Myung Guk Han

- EPI_ISL_412912 BetaCoV/Germany/Baden-Wuerttemberg-1/2020 Europe / Germany / Baden-Wuerttemberg 2020-02-25 State Health Office Baden-Wuerttemberg Charité Universitätsmedizin Berlin, Institute of Virology Victor M Corman, Julia Schneider, Barbara Mühlemann, Talitha Veith, Jörn Beheim-Schwarzbach, Terry Jones, Rainer Oehme, Silke Fischer, Christian Drosten

- EPI_ISL_412972 BetaCoV/Mexico/CDMX/InDRE_01/2020 North America / Mexico / Mexico City 2020-02-27 Instituto Nacional de Enfermedades Respiratorias Instituto de Diagnostico y Referencia Epidemiologicos (INDRE) Ramirez-Gonzalez Ernesto, Garces-Ayala Fabiola, Araiza-Rodriguez Adnan, Mendieta-Condado Edgar, Rodriguez-Maldonado Abril, Wong-Arambula Claudia, Vazquez-Perez Joel, Martinez Arturo, Boukadida Celia, Munoz-Medina Esteban, Sanchez Alejandro, Isa Pavel, Taboada Blanca, Lopez Susana, Arias Carlos, Barrera-Badillo Gisela, Hernandez-Rivas Lucia, Lopez-Martinez Irma

- EPI_ISL_412973 BetaCoV/Italy/CDG1/2020 Europe / Italy / Lombardy 2020-02-20 Department of Infectious Diseases, Istituto Superiore di Sanità, Roma , Italy Virology Laboratory, Scientific Department, Army Medical Center Paola Stefanelli, Stefano Fiore, Antonella Marchi, Eleonora Benedetti, Concetta Fabiani, Giovanni Faggioni, Antonella Fortunato, Riccardo De Santis, Silvia Fillo, Anna Anselmo, Andrea Ciammaruconi, Stefano Palomba, Florigio Lista

- EPI_ISL_412974 BetaCoV/Italy/SPL1/2020 Europe / Italy / Rome 2020-01-29 Department of Infectious Diseases, Istituto Superiore di Sanità, Rome, Italy Virology Laboratory, Scientific Department, Army Medical Center Paola Stefanelli, Stefano Fiore, Antonella Marchi, Eleonora Benedetti, Concetta Fabiani, Giovanni Faggioni, Antonella Fortunato, Silvia Fillo, Riccardo De Santis, Andrea Ciammaruconi, Giancarlo Petralito, Filippo Molinari, Florigio Lista

We gratefully acknowledge the Authors, the Originating and Submitting Laboratories for the sequence and metadata shared through ncbi database (https://www.ncbi.nlm.nih.gov/nucleotide/)

- NC_045512.2 Severe acute respiratory syndrome coronavirus 2 isolate Wuhan-Hu-1, complete genome.Dec-2019. Shanghai Public Health Clinical Center & School of Public Health, Fudan University, Shanghai, China. Wu,F., Zhao,S., Yu,B., Chen,Y.-M., Wang,W., Hu,Y., Song,Z.-G., Tao,Z.-W., Tian,J.-H., Pei,Y.-Y., Yuan,M.L., Zhang,Y.-L., Dai,F.-H., Liu,Y., Wang,Q.-M., Zheng,J.-J., Xu,L., Holmes,E.C. and Zhang,Y.-Z.

- MN908947 Severe acute respiratory syndrome coronavirus 2 isolate Wuhan-Hu-1, complete genome. Dec-2019. Shanghai Public Health Clinical Center & School of Public Health, Fudan University, Shanghai, China. Wu,F., Zhao,S., Yu,B., Chen,Y.-M., Wang,W., Hu,Y., Song,Z.-G., Tao,Z.-W., Tian,J.-H., Pei,Y.-Y., Yuan,M.L., Zhang,Y.-L., Dai,F.-H., Liu,Y, Wang,Q.-M., Zheng,J.-J., Xu,L., Holmes,E.C. and Zhang,Y.-Z.
